# microRNAs in viral oncogenesis

**DOI:** 10.1186/1742-4690-4-82

**Published:** 2007-11-24

**Authors:** Vinod Scaria, Vaibhav Jadhav

**Affiliations:** 1 GN Ramachandran Knowledge Center for Genome Informatics, Institute of Genomics and Integrative Biology, CSIR, Mall Road, Delhi, 110 007, India

## Abstract

MicroRNAs are a recently discovered class of small noncoding functional RNAs. These molecules mediate post-transcriptional regulation of gene expression in a sequence specific manner. MicroRNAs are now known to be key players in a variety of biological processes and have been shown to be deregulated in a number of cancers. The discovery of viral encoded microRNAs, especially from a family of oncogenic viruses, has attracted immense attention towards the possibility of microRNAs as critical modulators of viral oncogenesis. The host-virus crosstalk mediated by microRNAs, messenger RNAs and proteins, is complex and involves the different cellular regulatory layers. In this commentary, we describe models of microRNA mediated viral oncogenesis.

## Background

Interest in the involvement of infectious agents in oncogenic transformation, and more so viruses, has been of historical importance, probably starting with Rous' discovery of filterable particles that could transmit avian sarcoma [1]. This was followed by the discovery of the role of other viruses in oncogenic transformation of eukaryotic cells. Subsequently, attempts were made to understand the molecular mechanisms of viral oncogenesis. A new field of noncoding RNA mediated regulation has emerged following the discovery of microRNAs, which are ~22 nucleotide long noncoding regulatory RNAs found in eukaryotes and viruses, and the unraveling of their critical roles in normal and abnormal biological processes including development, host-virus interaction and neoplasia [2]. These small endogenous noncoding RNAs are derived from introns or intergenic regions in the genome, many of which were previously thought to be 'junk DNA'. They are processed from hairpin forming precursors by a battery of cellular proteins. These small RNAs, in association with a ribonucleoprotein complex termed as the RNA Induced Silencing Complex, or RISC, mediate post-transcriptional regulation of gene expression. They do this by binding to the 3'UTR regions of the transcripts, harboring regions of imperfect complementarity. The biogenesis and action of microRNAs have been extensively reviewed [3,4]. The role played by microRNAs in the defense of mammalian cells against virus infection has also been discussed recently [5-7].

**Figure 1:**
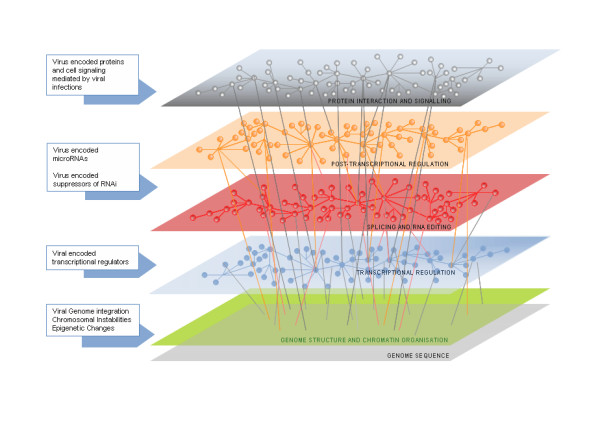
Model for host-virus crosstalk in viral oncogenesis. The planes describe the different layers of cellular regulatory organization and the interconnections between different layers marked by thin lines. The arrows on the left side show the mechanisms where viruses or virus encoded gene products interact or interfere with host regulatory mechanism.

MicroRNAs constitute a hitherto unexplored layer of genetic interactions between the virus and the host. The regulatory impact of microRNAs is huge because a single microRNA can regulate multiple transcripts and multiple microRNAs can regulate a single transcript. This is very similar to transcriptional regulatory networks. Models of microRNA in host-virus cross-talk have been reviewed recently [8,9]. The recent discovery of microRNAs encoded by a number of viruses, including many human oncogenic viruses, has attracted renewed interest in the molecular mechanism of viral oncogenesis. This novel regulatory layer, mediated by microRNAs, has a far-reaching impact on the latency and pathogenesis of viruses, including the mechanism of virus induced cancers. The molecular role of microRNAs in viral oncogenesis may be diverse, ranging from viral encoded microRNAs to virus encoded suppressors of RNA interference. Cancer itself is multifactorial, wherein deregulation at multiple levels culminates in the global regulatory derangement, thereby making molecular oncogenesis an enigma. In this review we discuss, in light of recent reports, the various possible mechanisms and/or models of host-virus interactions culminating in oncogenesis mediated by microRNAs. Figure 1 provides a simplistic overview of the role of microRNAs in viral oncogenesis. Challenges in the field and future perspectives are also discussed.

Here, we survey host-virus crosstalk culminating in oncogenesis encompassing five major models: (1) viral microRNAs and their effects, (2) viral integration and its effects on host and viral microRNAs, (3) virus induced genetic instabilities, (4) virus mediated suppression of RNA interference, and (5) Virus induced epigenetic changes.

### Virus encoded microRNAs and their cellular targets

Recent genome-wide screens, enabled by computational approaches and high-throughput validation, have discovered 109 microRNA precursors encoded by viruses. A major chunk of the currently known microRNAs are encoded by the herpes virus family of viruses which include a number of human oncogenic viruses like Herpes Simplex virus, Kaposi Sarcoma Herpes Virus and Epstein Barr virus. The logical question then would be: what are the targets of these virus encoded microRNAs and what are the physiological processes regulated by these microRNAs. A computational analysis of the targets of EBV encoded microRNAs, using a consensus prediction of three commonly used target prediction algorithms, reveals that the transcripts targeted by these microRNAs are over-represented in the genes associated with apoptosis and tumor-suppression [9]. Moreover, a majority of these RNAs are derived from the BART and BHRF cluster of genes, which are classically known to be activated during latent phase of the virus [10]. This finding becomes more relevant in light of recent evidence that suggests that in EBV induced gastric carcinoma, the BART cluster of microRNAs are expressed, while the BHRF cluster is not. Both together suggest an important role for the BART cluster of microRNAs in EBV mediated gastric carcinomas [11] and probably, in other cancers caused by the virus. Recent experimental evidence on the targets of Herpes Simplex Virus, another related Herpes virus, also shows that virus encoded microRNAs target transcripts involved in apoptosis [12]. Similarly another oncogenic virus in avians, Marek's Disease virus (MDV) has been recently shown to encode a microRNA targeting the latency associated transcript and its expression in MDV induced tumors [13,14].

Computational algorithms for prediction of miRNAs' target transcripts have improved drastically over recent years. The current state of the art computational techniques and their application in the prediction of microRNA-targets was reviewed by Maiere and Enright [15]. Efficient computational methods, combined with high-throughput experimental methods, have greatly facilitated the task of miRNA and target identification. The putative functional roles of virus encoded microRNAs are summarized in Table 1. However, the steady increase in the number of microRNAs encoded by viruses does not match with the number of targets experimentally validated, which is a deterrent towards understanding the functional role of these microRNAs. This is primarily because rapid experimental validation of computational predictions is still an unmet challenge.

**Table 1:** List of virus-encoded microRNAs and their possible functional roles.

**Virus Name**	**Family**	**Number of microRNA precursors**	**Functional role**
Epstein Barr virus	Herpesviridae	23	? Regulation of Tumor Suppression/Apoptosis
Herpes Simplex Virus 1	Herpesviridae	2	Regulation of Apoptosis
Human cytomegalovirus	Herpesviridae	11	
Kaposi sarcoma-associated herpesvirus	Herpesviridae	13	Regulation of cell adhesion, migration, and angiogenesis
Mareks disease virus (Type 1 and 2)	Herpesviridae	25	Virus-induced transformation of chicken T cells
Mouse gammaherpesvirus 68	Herpesviridae	9	-
Rhesus lymphocryptovirus	Herpesviridae	16	-
Rhesus monkey rhadinovirus	Herpesviridae	7	? Regulation of immune response pathways
Simian virus 40	Polyomaviridae.	1	Cytotoxic T-cell escape
Human immunodeficiency virus 1	Retroviridae	2	? Regulation of latency

### Virus integration modulating host microRNAs

Integration of the viral genome into the host and its effect in tumorigenesis have been active areas of research in oncology. This field has been particularly enriched by studies of gene therapy vectors and the emergence of transposon-mediated mutagenesis as tools to study gene function. Viral integrations can occur non-randomly in the host genome, with some classes of viruses showing specific insertion patterns. Viral integration is also known to result in short or long range effects on the expression of host genes including genes which code for microRNAs. Recent reports show that oncogenic microRNAs could be up-regulated by viral integration in their vicinity [16,17]. Feitelson *et al*. [18]reported that in Hepatitis B induced viral hepato-carcinogenesis a large number of viral integration events occur near or within fragile sites and/or other cancer-associated host loci which are prone to instability or are critical for tumor development and progression [19]. Similarly, loss of miRNA function can also occur via viral integration because some microRNAs fall within regions disrupted by viral integration. An example for this is hsa-mir-566, a repeat associated microRNA which falls in a retroviral integration site (unpublished results). This throws open a new avenue, whereby stochasticity of viral integration events could differentially modulate the expression of tightly regulated factors, culminating in neoplasia. The effect of viral integration and the consequential modulation of gene expression, including microRNAs and modulators of microRNA expression, have not been explored in detail. Recent analysis of chromosomal susceptibility loci in murine cancers has suggested the association between the locations of mouse miRNAs and known sites of retroviral integration in mouse cancers [20]. Computational analysis of viral integration site libraries for microRNA genes in nearby regions would prove to be useful in understanding viral modulation of microRNA expression and the pathogenesis of viral oncogenesis. Viral transcripts could also possibly modulate host microRNA expression by sequestering microRNAs or the cell's microRNA processing machinery, and tilting the balance of normal cellular regulation, similar to the description of target mimicry in Arabidopsis [21].

### Virus induced genetic instabilities and errors in DNA repair

Apart from viral integration, genetic instabilities induced by viruses have also been extensively studied. Fragile sites and genomic instabilities, including aneuploidies, have been of particular interest in studying mechanisms of oncogenesis. Recent computational screens for fragile chromosomal breakpoints associated with cancers, including regions of instability induced by papillomaviruses, have shown that many microRNAs including oncogenic microRNAs lie in close proximity to regions of chromosomal rearrangements [19]. Apart from chromosomal rearrangements and instability, virus-mediated suppression of cellular DNA repair, activation of telomerase and telomere maintenance have been well explored in cancers. Examples of viral supressors of the DNA repair mechanism include the E6 protein of Human Papillomavirus which down regulates the methyl guanine methyltransferase (MGMT) and the X protein encoded by Hepatitis B virus, which interacts with UVDDB, a putative DNA repair protein. Viral activation of telomerase has been well studied in the context of KSHV where KSHV viral associated nuclear antigen which binds to Sp1 and transactivates telomerase expression [22]. Widespread mutations in the host genome and their effects on microRNA mediated regulation have not been actively pursued though accounts of mutational effects on microRNAs and target regions in the 3'UTR have emerged very recently [23].

### Virus encoded suppressors of RNAi

RNA interference has emerged as a mechanism of antiviral defense in many plants and insects. Viruses have overcome this by encoding for proteins that can particularly suppress the RNAi mechanism by multiple methods ranging from binding to dsRNA, to binding and disrupting functions of key proteins involved in microRNAs processing [24-26]. Such global suppressions of host microRNA expression have been recently shown in HIV infection studies [27]. Recently Haasnoot *et al*. [28] have shown that Ebola Virus VP35 protein is a suppressor of RNAi, akin to the function of Tat in HIV infection. This means that suppressors of RNAi are a conserved feature in many pathogenic viruses. Many of these mechanisms culminate in deregulation of microRNAs biogenesis. Such global derangement of microRNA biogenesis has been recently shown to be oncogenic [29]. Direct evidence for virus encoded suppressors of RNAi resulting in a global derangement of microRNA biogenesis resulting in abnormal microRNAs mediated regulation of key tumor suppressors and cell cycle checkpoint genes remains to be established. It would also be interesting to explore how viral microRNAs modulate the cellular RNAi mechanism to regulate viral and/cellular targets.

### Virus induced epigenetic changes in the host

Epigenetic changes have recently been shown to be critical in modulating the spatial and temporal expression profiles of microRNAs. Viruses, especially those involved in oncogenesis have been extensively investigated for their potential to modulate host epigenetic changes, including DNA methylation, histone modification and chromatin remodeling. Flanagan has exhaustively reviewed the different models of host epigenetic regulation by oncogenic viruses [30]. The possibility of viral proteins to modulate microRNA expression through epigenetic mechanisms has not been thoroughly studied. Recent evidence has substantiated an epigenetic role for viral microRNA in the transcriptional silencing of HIV [31]. Further understanding of how viral microRNAs modulate epigenetic regulation would open up potential new arenas for therapy.

### Virus infection modulating microRNA expression and host signaling

Viral infections have been shown to modulate host gene expression in multiple ways. One major pathway used by host cells in viral defense is the Toll-like receptor (TLR) pathway. Viruses have the potential to activate Toll-like receptors. TLR-pathways can trigger a cascade of downstream effectors, some leading to the activation of transcriptional modulators such as NF kappa B which can in turn regulate the expression of oncogenic microRNAs [32]. It remains to be seen whether this type of virus-initiated circuitry contributes substantively to the effects of chronic viral infections which can result in cancers.

Separately, recent evidence suggests that HIV-1 infection can significantly remodel the host cell's microRNA profile [33]. Specifically, HIV-1 appears to down regulates a number of antiviral microRNA genes and to up regulates of a small number of microRNAs, including the miR-17-92 cluster of microRNAs previously known to be involved in oncogenesis [27]. The exact role of these microRNAs in viral pathogenesis and/latency is not known. There is a possibility that the functional role of the microRNAs would be different in different cell-types due to transcript diversity between cell types.

### The way forward- understanding microRNA role in viral pathogenesis: a systems biology approach to host-virus interaction

The current understanding of the role of microRNAs in host-virus crosstalk or viral oncogenesis is far from complete. There is a need to co-ordinate efforts from multiple experimental labs to build a holistic view of host-virus interactions. This would include prediction and validation of genome-scale protein-protein and microRNA -target interactions, along with temporal analysis of gene expression which could be integrated onto a bioinformatics platform to understand the dynamics and intricacies of host-virus crosstalks. Recently, a number of databases of biological pathways and protein interactions including host-pathogen interactions as in the case of HIV have been developed by Reactome [34]. Similarly, there have been consistent efforts to collect gene expression and proteomic datasets in central repositories [35]. Availability of high-throughput expression and proteomics coupled to high performance operating platforms could allow one to integrate questions and answers in a systems biology manner. This collective approach could greatly aid in understanding host-virus interactions in an inclusive way.

## Authors' contributions

VJ and VS conceived the topic. Both authors discussed the data and formulated the models .VS wrote the manuscript. Both authors read and approved the final manuscript.
